# The Predictive Value of Integrated Pulmonary Index after Off-Pump Coronary Artery Bypass Grafting: A Prospective Observational Study

**DOI:** 10.3389/fmed.2017.00132

**Published:** 2017-08-09

**Authors:** Evgenia V. Fot, Natalia N. Izotova, Anjelika S. Yudina, Aleksei A. Smetkin, Vsevolod V. Kuzkov, Mikhail Y. Kirov

**Affiliations:** ^1^Department of Anesthesiology and Intensive Care Medicine, Northern State Medical University, Arkhangelsk, Russia

**Keywords:** postoperative respiratory failure, coronary artery bypass grafting, monitoring, microstream capnography, integrated pulmonary index

## Abstract

**Background:**

The early warning scores may increase the safety of perioperative period. The objective of this study was to assess the diagnostic and predictive role of Integrated Pulmonary Index (IPI) after off-pump coronary artery bypass grafting (OPCAB).

**Materials and Methods:**

Forty adult patients undergoing elective OPCAB were enrolled into a single-center prospective observational study. We assessed respiratory function using IPI that includes oxygen saturation, end-tidal CO_2_, respiratory rate, and pulse rate. In addition, we evaluated blood gas analyses and hemodynamics, including ECG, invasive arterial pressure, and cardiac index. The measurements were performed after transfer to the intensive care unit, after spontaneous breathing trial and at 2, 6, 12, and 18 h after extubation.

**Results and Discussion:**

The value of IPI registered during respiratory support correlated weakly with cardiac index (rho = 0.4; *p* = 0.04) and ScvO_2_ (rho = 0.4, *p* = 0.02). After extubation, IPI values decreased significantly, achieving a minimum by 18 h. The IPI value ≤9 at 6 h after extubation was a predictor of complicated early postoperative period (AUC = 0.71; *p* = 0.04) observed in 13 patients.

**Conclusion:**

In off-pump coronary surgery, the IPI decreases significantly after tracheal extubation and may predict postoperative complications.

## Introduction

Cardiac surgery can be complicated by respiratory failure that may contribute to increased morbidity and additional health-care costs (1, 2). The outcome of coronary artery bypass grafting can be significantly influenced by decompensation caused by chronic pulmonary diseases and other complications (atelectases, pleuritis, etc.) (3–5). Therefore, the thorough postoperative monitoring of pulmonary function during both mechanical ventilation and spontaneous breathing may be of a great value. Notably, the modern monitoring devices should be accurate and non- or minimally invasive with measurements that are continuous and results easily interpreted (6).

To maintain respiratory function, the cardiosurgical patients are monitored using pulse oximetry, capnography, respiratory rate, and discrete blood gas analysis (7–9). Although blood gas analyses are the gold standard for early detection of different types of respiratory failure, they are invasive, cannot be measured continuously, and frequently impose a delay between sampling and availability of results (10). Thus, the early warning systems allowing early recognition of critical respiratory events might be of value when patient is monitored both in the intensive care unit (ICU), postoperative ward, and high dependency unit. This approach can be particularly useful with a limited number of medical staff. Several observational studies indicate that early warning systems improve detection of complications (11), and their use is recommended by the World Federation of Societies of Anesthesiologists to facilitate the work of nurses and physicians in the ICU (12, 13).

The Integrated Pulmonary Index (IPI) is an automated value calculated by one monitor (Capnostream-20, Medtronic, Israel) and can be considered as an automated early warning system. The IPI algorithm utilizes the real time measures and interactions of four parameters—end-tidal CO_2_ (PetCO_2_), respiration rate, pulse rate, and oxygen saturation (SpO_2_) to provide an assessment of the patient’s respiratory status. The calculation of the IPI is based on the fuzzy logic principle, a mathematical model, which mimics human logic thinking; detailed description of the algorithm was provided by Ronen et al. (14) The values of IPI below 7 have been suggested to be an indicator for respiratory deterioration (14).

Currently, only few investigations of IPI were performed during non-cardiosurgical procedures (14–18) In these studies, IPI algorithm correlated with the respiratory status and has demonstrated the ability for promoting early awareness to changes in a patient’s respiratory system.

The aim of our study was to assess the diagnostic and predictive role of IPI during the discontinuation from mechanical ventilation and in the early postextubation period after off-pump coronary artery bypass grafting (OPCAB).

## Materials and Methods

The study was performed in a 900-bed university hospital (City Hospital #1 of Arkhangelsk, Russia). During 2015, 40 adult patients undergoing elective OPCAB were enrolled into an observational prospective study. The study design and the informed consent form were approved by the Ethical Committee of Northern State Medical University (Arkhangelsk, Russian Federation) and registered with http://ClinicalTrials.gov (ref: NCT02524522). Written informed consent was obtained from every patient. Exclusion criteria were age <18 and >80 years, morbid obesity with body mass index >40 kg/m^2^, and constant atrial fibrillation.

All patients were intubated using the standard induction technique with sodium thiopental (4 mg/kg), fentanyl (2.5–3.0 μg/kg) and pipecuronium bromide (0.1 mg/kg). Anesthesia was maintained using sevoflurane (0.5–3.0 vol.% at the end of expiration) and fentanyl (2.0–4.0 μg/kg/h). Depth of anesthesia was adjusted to maintain BIS values between 40 and 60 (LifeScope, Nihon Kohden, Japan).

In all cases, preoxygenation with 80% O_2_ was provided during 3–5 min before anesthesia. After tracheal intubation, patients were ventilated using a protective volume-controlled mode (Dräger Primus, Germany) with tidal volume of 6–8 mL/kg of predicted body weight, flow of 1 L/min and positive end-expiratory pressure (PEEP) of 5 cm H_2_O. FiO_2_ was set to at least 50% or higher to achieve intraoperative SpO_2_ above 95%. The respiratory rate was adjusted to maintain PetCO_2_ value within 30–35 mmHg.

After surgery, all patients were transferred to the postoperative cardiac ICU and shortly sedated with continuous infusion of propofol (2–4 μg/kg/h) to maintain BIS values within 60–70. Respiratory support in ICU was provided by a G5 ventilator (Hamilton Medical, Switzerland) using pressure controlled ventilation mode with parameters of intraoperative ventilation. Additionally, all patients received recruitment maneuver by raising the PEEP to 20 cm H_2_O for 5 min.

After the initial measurements, sedation was stopped, and the weaning from respiratory support was initiated. The weaning protocol included gradual reduction of inspiratory pressure and mandatory respiratory rate, as well as spontaneous breathing trial. After passing the 30-min spontaneous breathing trial, all the patients were immediately extubated. After extubation, the patients received a supplementary oxygen flow of 4 L/min *via* a nasal catheter. During the weaning process and in the early postextubation period, all the patients received continuous infusion of fentanyl and discrete administration of paracetamol for multimodal analgesia. In addition, the postoperative therapy included aspirin, low-molecular weight heparins, and bisoprolol.

The measurements included ventilator parameters, blood gas analyses (ABL800Flex, Radiometer, Denmark), PetCO_2_, SpO_2_, respiratory rate, pulse rate, and IPI (Capnostream-20, Medtronic). The IPI measurement is based on continuous transformation of SpO_2_, PetCO_2_, pulse rate, and respiratory rate values into a single index from 1 to 10, where “10” indicates a normal respiratory status, and “1” indicates that patient requires immediate intervention. We distributed patients into two subgroups: with optimal (IPI 9–10) and suboptimal (IPI ≤ 8) IPI values (Table 1). After tracheal extubation, for a more accurate assessment of the IPI, all the values were measured following breathing during 5 min without supplemental oxygen (FiO_2_ 0.21), avoiding the reduction of SpO_2_ less than 88%. Continuous hemodynamic measurements included ECG monitoring, invasive arterial pressure and cardiac output measured with pulse wave transit time (esCCO, Nihon Kohden, Japan).

**Table 1:** The clinical interpretation of Integrated Pulmonary Index (IPI) (14).

IPI	Patient status	Subgroups
10–9	Normal	Optimal values
8	Within normal range	Suboptimal values
7	Close to normal range; requires attention	
5–6	Requires attention and may require intervention	
3	Requires intervention	
1–2	Requires immediate intervention	

All these parameters were registered after transfer to the ICU, as well as after spontaneous breathing trial and at 2, 6, 12, and 18 h after extubation. In addition, we recorded the preoperative EuroScore II, perioperative fluid balance, left ventricle ejection fraction assessed by transthoracic echocardiography before and 24 h after surgery, duration of postoperative mechanical ventilation and ICU stay, as well as early postoperative complications and hospitalization time. Postoperative complications were assessed according to the categories as predefined the study protocol: arrhythmias, hemorrhage, respiratory complications, neurological complications, and postoperative myocardial damage. Arrhythmic complications were comprised of any episode of atrial fibrillation, ventricular arrhythmia, or fibrillation requiring therapeutic intervention. Hemorrhagic complications were defined as drainage blood loss of more than 200 mL/h for three consecutive hours or re-sternotomy. Respiratory complications were reintubation, need for prolonged oxygen therapy, pneumothorax, hydrothorax, chylothorax, or pneumonia. Patients were considered as requiring prolonged oxygen therapy after extubation in case if needed oxygen insufflation more than 12 h to maintain SpO_2_ > 93%. Neurological complications were defined as postoperative delirium or stroke. The postoperative myocardial damage was defined as an increase in the plasma concentration of creatine kinase-MB > 50 pg/mL.

### Statistical Analysis

For data collection and analysis, we used SPSS software (version 17.0; SPSS Inc., USA) and MedCalc software (version 12.3, MedCalc Software, Belgium). Due to pilot design of the study, the sample size was limited by 40 patients. All the variables were expressed as median (25^th^–75^th^ interquartile interval). The groups were compared using Mann–Whitney test. The intragroup comparisons were performed by Friedman and *post hoc* Wilcoxon tests with Bonferroni correction. For correlation analysis, we used Spearman test. Nominal data were compared using χ^2^ test and expressed as patient number. To evaluate the ability of IPI and PetCO_2_ to predict cardiac index <2.5 L/min/m^2^ during mechanical ventilation, we performed ROC-curve analysis and calculated area under the ROC curve (AUC). The ROC analysis was also used to assess the capability of IPI and PaO_2_/FiO_2_ measured at 6 h after extubation for prediction of postoperative complications during 24 h. The optimal cutoff point for IPI was determined by maximum value of the Youden Index (maximizing sensitivity and specificity). For *post hoc* intragroup comparisons, *p* value < 0.013 was considered as statistically significant. In all other cases, *p* value < 0.05 was regarded as statistically significant.

## Results

We enrolled 30 males and 10 females. Demographic and baseline characteristics of the patients, as well as postoperative complications are shown in Table 2.

**Table 2:** The patient characteristics during perioperative period.

Characteristics	Value
Age, years	62 (55–70)
BMI, kg/m^2^	30 (27–31)
EuroScore II, points	1.15 (0.85–1.59)
Duration of surgery, min	210 (185–250)
Grafts, number	3 (2–4)
Intraoperative fluid balance, mL	900 (563–1,238)
**Baseline characteristics after admission to the ICU**	
IPI	9 (8–10)^a^
PaO_2_/FiO_2_, mmHg	270 (193–332)
SpO_2_, %	100 (98–100)
PetCO_2_, mmHg	30 (28–34)
PR, bpm	61 (54–75)
RR/min	15 (13–15)
PaCO_2_, mmHg	39 (36–41)
Cardiac index, L/min/m^2^	2.41 (2.04–2.76)
Duration of postoperative ventilation, min	193 (138–258)
Duration of ICU stay, h	24 (24–66)
**Postoperative complications (*n* = 13)**	
Arrhythmia	5
Respiratory complications	6
Hemorrhagic complications	1
Neurological complications	1
Myocardial damage	0

*^a^n = 39; in all other cases n = 40*.

*Data presented as median (25th–75th percentile), percentage or numbers*.

*BMI, body mass index; PR, pulse rate; RR, respiratory rate; ICU, intensive care unit; IPI, Integrated Pulmonary Index*.

**Figure 1:**
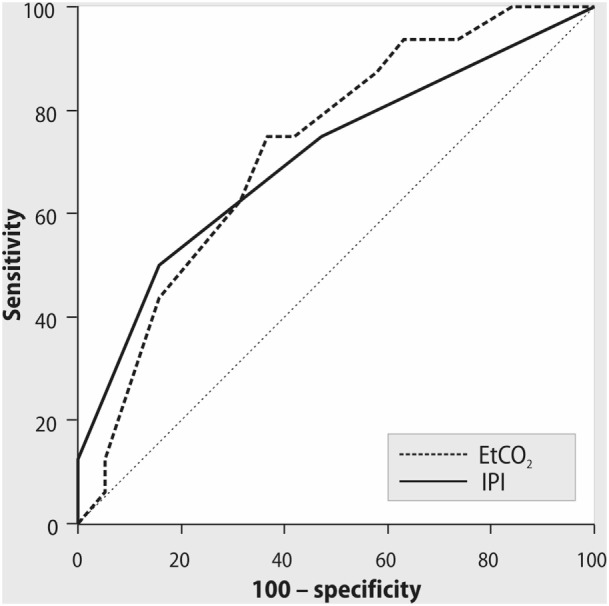
ROC curves for Integrated Pulmonary Index (IPI), end-tidal CO_2_, and cardiac index < 2.5 L/min/m^2^ during mechanical ventilation. AUC = 0.72, *p* = 0.02; cutoff point of IPI ≤ 8, with sensitivity 84%, specificity 53%, positive predictive value 64%, negative predictive value 75%. AUC = 0.73, *p* = 0.02; cutoff point of PetCO_2_ ≤ 30 mmHg, with sensitivity 78%, specificity 68%, positive predictive value 70%, negative predictive value 76%.

After admission to the ICU, we had difficulties in registration of the IPI value only in one patient. Notably, 10 min later IPI was registered in 100% of patients. After admission to the ICU, 5% of patients required attention according to their respiratory status and had IPI < 7. Simultaneously, 63% of patients had PaO_2_/FiO_2_ < 300 mmHg. The IPI values, measured after ICU admission, weakly correlated with cardiac index (rho = 0.4, *p* = 0.04) and ScvO_2_ (rho = 0.4, *p* = 0.02). The decreased values of IPI and PetCO_2_ during controlled mechanical ventilation were associated with CI < 2.5 L/min/m^2^ (cutoff point for IPI ≤ 8, sensitivity 84%, specificity 53%, positive predictive value 64%, negative predictive value 75%, AUC = 0.72, *p* = 0.02; cutoff point for PetCO_2_ ≤ 30 mmHg, sensitivity 78%, specificity 68%, positive predictive value 70%, negative predictive value 76%, AUC = 0.73, *p* = 0.02, Figure 1).

**Figure 2:**
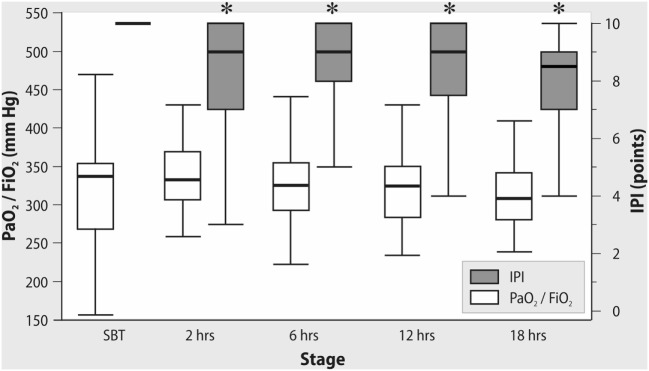
Changes in PaO_2_/FiO_2_ and Integrated Pulmonary Index (IPI) after tracheal extubation. *Wilcoxon test, *p* < 0.01.

**Figure 3:**
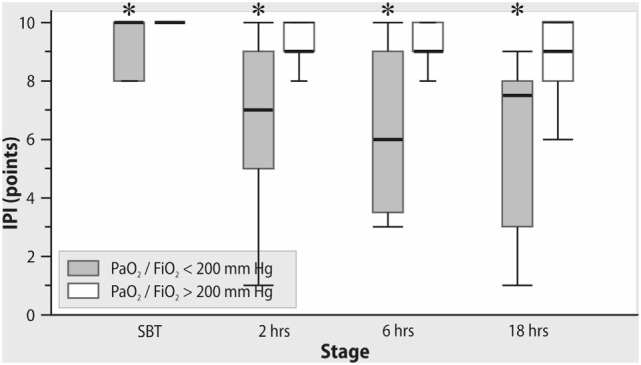
The changes in Integrated Pulmonary Index (IPI) after extubation in subgroups with PaO_2_/FiO_2_ <200 mmHg and >200 mmHg on admission to the intensive care unit. *Mann–Whitney test, *p* < 0.05.

All patients were successfully weaned from mechanical ventilation. PaO_2_/FiO_2_ ratio was stable both during the spontaneous breathing trial and after tracheal extubation. In contrast, IPI decreased significantly after OPCAB with a minimal value at 18 h after extubation (Figure 2). As shown in Figure 3, in patients with PaO_2_/FiO_2_ < 200 mmHg on ICU admission, the IPI values at 2, 6, 12, and 18 h after extubation did not exceed suboptimal range (≤8) (*p* < 0.05 as compared to IPI values of the subgroup with PaO_2_/FiO_2_ > 200 mmHg).

In addition, the suboptimal IPI values at 2 h after tracheal extubation were associated with higher preoperative EuroScore and decreased left ventricular ejection fraction before and after OPCAB (Table 3). In the subgroup with IPI ≤ 8, we observed decreased SpO_2_ and etCO_2_, as well as increased pulse rate. Higher IPI values were associated with positive fluid balance and decreased rate of diuretic administration at the first day of ICU stay.

**Table 3:** Comparative characteristics in subgroups of patients with optimal (>8) and suboptimal (≤8) IPI values at 2 h after extubation.

Characteristics	IPI_optimal_ (*n* = 25)	IPI_suboptimal_ (*n* = 13)	*p*-Value
Age, years	63 (55–70)	65 (56–74)	0.69
BMI, kg/m^2^	29 (27–32)	30 (28–32)	0.63
EuroScore II, points	1.01 (0.84–1.5)	1.4 (1.2–2.05)^a^	**0.03**
EF before surgery, %	60 (55–66)	52 (46–60)^a^	**0.02**
EF after surgery, %	63 (60–68)	57 (52–62)^a^	**0.007**
SpO_2_, %	95 (93–98)	93 (89–95)^a^	**0.045**
etCO_2_, mmHg	37 (35–39)	33 (30–35)^a^	**0.03**
PR, bpm	77 (70–88)	88 (75–99)^a^	**0.04**
RR/min	14 (14–18)	15 (15–18)	0.33
PaO_2_/FiO_2_, mmHg	324 (301–349)	317 (293–331)	0.32
PaCO2, mmHg	38 (36–39)	36 (31–39)	0.58
Fluid balance, mL	320 (−110 to 498)	−225 (−337 to +275)^a^	**0.03**
Urine output, mL/kg/h	1.0 (0.7–1.3)	1.2 (1.0–1.6)	0.06
Administration of diuretics	4	11^b^	**0.05**
Duration of surgery, min	195 (172–237)	245 (202–255)	0.13
Duration of ICU stay, h	24 (24–72)	24 (24–48)	0.30
Hospitalization time, days	9 (7–10)	9 (8–12)	0.35

*Data presented as median (25th–75th percentile), percentage or numbers*.

*BMI, body mass index; EF, ejection fraction; PR, pulse rate; RR, respiratory rate; ICU, intensive care unit*.

*Bold font indicates statistical significance with p < 0.05*.

*^a^Mann–Whitney test, p < 0.05*.

*^b^χ^2^, p < 0.05*.

**Figure 4:**
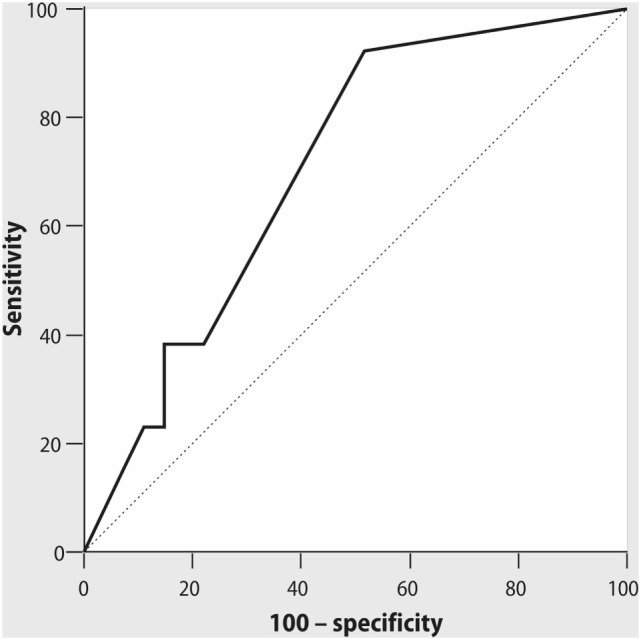
ROC curve for Integrated Pulmonary Index (IPI), measured at 6 h after tracheal extubation, and early postoperative complications. AUC = 0.71; *p* = 0.04, cutoff point of IPI ≤ 9 with sensitivity 92% and specificity 48%, positive predictive value 57%, negative predictive value 89%.

The length of ICU and hospital stay did not differ between the patients with optimal and suboptimal IPI values. We did not find any associations between PaO_2_/FiO_2_ ratio and the length of ICU stay either. However, IPI value ≤9 at 6 h after extubation demonstrated moderate predictive ability for early postoperative complications (AUC = 0.707; *p* = 0.04, with sensitivity 92% and specificity 48%, positive predictive value 57%, negative predictive value 89%, Figure 4). PaO_2_/FiO_2_ ratio at 6 h after extubation did not demonstrate any predictive ability for postoperative complications (AUC = 0.543; *p* = 0.67).

## Discussion

Our study has shown that IPI can provide important information about respiratory and hemodynamic status of the cardiosurgical patient, especially during the postextubation period.

In our study, we observed difficulties in registration of the IPI value after admission to the ICU in one patient from 40 enrolled into the study; this problem can be explained by decreased perfusion, leading to low SpO_2_ signal. Low perfusion as well as motion artifacts are the well-known limitations of pulse oximetry observed in the early postoperative period after cardiac surgery (19).

After admission to the ICU, the number of patients with compromised respiratory function according to their PaO_2_/FiO_2_ values (63%) was higher than the number of patients requiring attention according to the IPI values (5%). During controlled mechanical ventilation, several components of IPI like respiratory rate, SpO_2_, and PetCO_2_, are determined mainly by the operator-depending settings of the ventilator that may not reflect the complex respiratory status. The association of IPI, measured after admission to the ICU, with cardiac index and ScvO_2_, observed in our study can be explained by the relationship between cardiac output, PetCO_2_, and oxygen transport (20). Although the described correlations were weak that can be caused by dependence of end-tidal CO_2_ not only from cardiac output but also from ventilation, metabolism, and other factors, our findings are consistent with other investigations in this field. In several studies, authors demonstrated that PetCO_2_ and cardiac output had a positive association in different categories of patients (21–23). Thus, Baraka and colleagues have shown that cardiac output correlated with PetCO_2_ during partial cardiopulmonary bypass and following weaning from bypass (22). In this study, PetCO_2_ > 30 mmHg during partial bypass predicted an adequate cardiac output after perfusion. At the same time, PetCO_2_ < 30 mmHg may correctly denote a low cardiac output only in combination with low ScvO_2_ (22). This relationship between PetCO_2_ and cardiac function can be relevant not only for cardiac surgery; thus, Dunham and colleagues have found that a decline in PetCO_2_ correlates with decrease in non-invasive cardiac output in emergently intubated trauma patients (23). Notably, the addition of pulse rate into the algorithm for calculation of IPI could improve the ability of this parameter to predict decreased cardiac output compared with PetCO_2_ alone. However, our ROC analysis has shown equal AUC to predict CI < 2.5 L/min/m^2^ both for IPI < 8 and for PetCO_2_ < 30 mmHg. The possible explanation for this finding could be that the heart rate is just one of the determinants of cardiac output, thus PetCO_2_ alone may have similar accuracy with IPI in predicting cardiac output after OPCAB.

Notably, reduced IPI values during controlled mechanical ventilation observed in our study can be explained by decreased PetCO_2_ levels. During spontaneous breathing with ambient air (FiO_2_ 21%), suboptimal IPI was also associated with decreased SpO_2_ values and increased pulse rate, aiming to maintain adequate cardiac output and oxygen delivery. We suppose that, summarizing the key cardiovascular and respiratory parameters, IPI can be a useful tool for postoperative assessment of patient in addition to PaO_2_/FiO_2_ ratio, which has a limited value due to dependence on FiO_2_ (24) This can explain the stable values of PaO_2_/FiO_2_ with simultaneous reduction of SpO_2_ and IPI after extubation while breathing with ambient air. It is important to mention that the measurement of IPI does not replace postoperative blood gases but it can potentially reduce the number of blood gas samples, is continuous as compared to discrete blood gases and can serve as a “monitoring bridge” after discontinuation of mechanical ventilation and invasive monitoring.

The association of suboptimal IPI values with preoperative EuroScore and ejection fraction before and after intervention demonstrates the relationship of IPI and severity of cardiac comorbidities. Several studies have shown that decreased ejection fraction after cardiac surgery may be associated with risk of sepsis, postoperative respiratory failure and prolonged mechanical ventilation (25, 26). Thus, the reduction of IPI after cardiac surgery can detect patients who require more complex hemodynamic monitoring and optimization including fluids, diuretics, inotrope/vasopressor support, and other therapies.

Association between IPI value ≤9, recorded at 6 h after extubation and the incidence of early complications after OPCAB seems to be relevant for prediction of the course of postoperative period. We did not find in other studies the data about the opportunity of IPI to predict the course of postoperative period, although IPI was effective in detection of clinically significant events, such as hypoxia or bradypnea, during the intraoperative period (16, 17). The complications observed during our study (predominantly, atrial fibrillation and respiratory failure) are accompanied by changes in respiratory and hemodynamic status of the patient. The patients after cardiac surgery can have a higher alert threshold of IPI as compared to other settings where the attention is required when IPI is ≤7. The diagnostic capabilities of IPI need further validation and studies including the assessment of IPI as a marker for the safe transfer from ICU.

### Study Limitations

Our findings have a limitation due to relatively small sample size. In addition, all the patients from our study received bisoprolol postoperatively that may influence the heart rate, as well as the IPI value.

## Conclusion

Integrated pulmonary index is associated with changes in cardiac output and may predict the postoperative complications during the discontinuation from mechanical ventilation and in the early postextubation period after OPCAB. This index may be a valuable adjunct to the routine monitoring during spontaneous breathing, but not during controlled mechanical ventilation.

## Ethics Statement

This study was carried out in accordance with the recommendations of the ethics committee of the Northern State Medical University (Arkhangelsk, Russian Federation). All subjects gave written informed consent in accordance with the Declaration of Helsinki. The protocol was approved by the ethics committee of the Northern State Medical University (Arkhangelsk, Russian Federation).

## Author Contributions

All authors had contributed equally.

## Conflict of Interest Statement

The study has received the research grant from Medtronic (Boulder, CO, USA). The authors declare that the research was conducted in the absence of any other commercial or financial relationships that could be construed as a potential conflict of interest.

## References

[1] Ng CS Wan S Yim AP Arifi AA Pulmonary dysfunction after cardiac surgery. Chest (2002) 4:1269–77. 10.1378/chest.121.4.1269 11948063

[2] Weissman C Pulmonary complications after cardiac surgery. Semin Cardiothorac Vasc Anesth (2004) 8:185–211. 10.1177/108925320400800303 15375480

[3] Forouzannia SK Abdollahi MH Mirhosseini SJ Hadadzadeh M Hosseini H Moshtaghion SH Perioperative predictors and clinical outcome in early and late ICU discharge after off-pump coronary artery bypass surgery. Acta Med Iran (2011) 49:307–9. 21713750

[4] Ho CH Chen YC Chu CC Wang JJ Liao KM Postoperative complications after coronary artery bypass grafting in patients with chronic obstructive pulmonary disease. Medicine (Baltimore) (2016) 95:1–5. 10.1097/MD.0000000000002926 PMC4779036 26937939

[5] Vakili M Shirani S Paknejad O Yousefshahi F. Acute respiratory distress syndrome diagnosis after coronary artery bypass: comparison between diagnostic criteria and clinical picture. Acta Med Iran (2015) 53:51–6. 25597606

[6] Vincent JL Rhodes A Perel A Martin GS Della Roca G Vallet B Clinical review: update on hemodynamic monitoring-a consensus of 16. Crit Care (2011) 15:1–8. 10.1186/cc10291 PMC3387592 21884645

[7] Brochard L Martin GS Blanch L Pelosi P Belda FJ Jubran A Clinical review: respiratory monitoring in the ICU – a consensus of 16. Crit Care (2012) 16:1–14. 10.1186/cc11146 22546221 PMC3681336

[8] Heinze H Sedemund-Adib B Heringlake M Meier T Eichler W. Relationship between functional residual capacity, respiratory compliance, and oxygenation in patients ventilated after cardiac surgery. Respir Care (2010) 55:589–94. 20420730

[9] MacNaughton PD Assessement of lung function in the ventilated patients. Intensive Care Med (1997) 8:810–8. 10.1007/s001340050417 9310798

[10] Technology Subcommittee of the Working Group on Critical Care, Ontario Ministry of Health. Noninvasive blood gas monitoring: a review for use in the adult critical care unit. CMAJ (1992) 146:703–12. 1562943 PMC1488288

[11] Alam N Hobbelink EL Tienhoven AJ The impact of the use of the early warning score (EARLY WARNING SYSTEMS) on patient outcomes: a systematic review. Resuscitation (2014) 85:587–94. 10.1016/j.resuscitation.2014.01.013 24467882

[12] Dawes TR Cheek E Bewick V Dennis M Duckitt RW Walker J Introduction of an electronic physiological early warning system: effects on mortality and length of stay. Br J Anaesth (2014) 4:603–9. 10.1093/bja/aeu107 24878563

[13] Kyriacos U Jelsma J James M Jordan S. Early warning scoring systems versus standard observations charts for wards in South Africa: a cluster randomized controlled trial. Trials (2015) 16:103–18. 10.1186/s13063-015-0624-2 25872794 PMC4374204

[14] Ronen M Weissbrod R Overdyk FJ Ajizian S Smart respiratory monitoring: clinical development and validation of the IPI™ (integrated pulmonary index) algorithm. J Clin Monit Comput (2017) 31:435–42. 10.1007/s10877-016-9851-7 26961501 PMC5346135

[15] Berkenstadt H Ben-Menachem E Herman A Dach R. An evaluation of the integrated pulmonary index (IPI) for the detection of respiratory events in sedated patients undergoing colonoscopy. J Clin Monit Comput (2012) 26:177–81. 10.1007/s10877-012-9357-x 22454276

[16] Garah J Adiv OE Rosen I Shaoul R. The value of integrated pulmonary index (IPI) monitoring during endoscopies in children. J Clin Monit Comput (2015) 29:773–8. 10.1007/s10877-015-9665-z 25666393

[17] Sabbatani P Mantovan R Electrical cardioversion of atrial fibrillation: evaluation of sedation safety with midazolam by means of EtCO_2_ and IPI algorithm analysis. Int J Cardiol (2013) 169:430–2. 10.1016/j.ijcard.2013.10.015 24157233

[18] Karbing DS Rees SE Jaffe MB. Journal of clinical monitoring and computing 2015 end of year summary: respiration. J Clin Monit Comput (2016) 30:7–12. 10.1007/s10877-015-9820-6 26719297

[19] Nitzan M Romem A Koppel R. Pulse oximetry: fundamentals and technology update. Med Devices (Auckl) (2014) 7:231–9. 10.2147/MDER.S47319 25031547 PMC4099100

[20] Isserles SA Breen PH. Can changes in end-tidal PCO_2_ measure changes in cardiac output? Anesth Analg (1991) 6:808–14. 1952183 10.1213/00000539-199112000-00023

[21] Whitaker DK Benson JP. Capnography standards for outside the operating room. Curr Opin Anaesthesiol (2016) 29(4):485–92. 10.1097/ACO.0000000000000355 27218421

[22] Baraka AS Aouad MT Jalbout MI Kaddoum RN Khatib MF Haroun-Bizri ST. End-tidal CO_2_ for prediction of cardiac output following weaning from cardiopulmonary bypass. J Extra Corpor Technol (2004) 36:255–7. 15559744

[23] Dunham CM Chirichella TJ Gruber BS Ferrari JP Martin JA Luchs BA In emergently ventilated trauma patients, low end-tidal CO_2_ and low cardiac output are associated and correlate with hemodynamic instability, hemorrhage, abnormal pupils, and death. BMC Anesthesiol (2013) 13:20–8. 10.1186/1471-2253-13-20 24020798 PMC3846857

[24] Karbing DS Kjaergaard S Smith BW Espersen K Allerød C Andreassen S Variation in the PaO_2_/FiO_2_ ratio with FiO_2_: mathematical and experimental description, and clinical relevance. Crit Care (2007) 11:R118. 10.1186/cc5697 17988390 PMC2246207

[25] Cohen AJ Katz MG Frenkel G Medalion B Geva D Schachner A. Morbid results of prolonged intubation after coronary artery bypass surgery. Chest (2000) 6:1724–31. 10.1378/chest.118.6.1724 11115465

[26] Topkara VK Cheema FH Kesavaramanujam S Mercando ML Cheema AF Namerow PB Coronary artery bypass grafting in patients with low ejection fraction. Circulation (2005) 112:1344–50. 10.1161/CIRCULATIONAHA.104.526277 16159844

